# Severe Gastritis Due to Nivolumab Treatment of a Metastatic Melanoma Patient

**DOI:** 10.3390/diagnostics12112864

**Published:** 2022-11-18

**Authors:** George Samonis, Aikaterini Bousmpoukea, Aristea Molfeta, Antonios D. Kalkinis, Kalliopi Petraki, Christos Koutserimpas, Dimitrios Bafaloukos

**Affiliations:** 1 First Oncology Department, Metropolitan Hospital, Neon Faliron, 18547 Athens, Greece; 2 Department of Nuclear Medicine, Metropolitan Hospital, Neon Faliron, 18547 Athens, Greece; 3 Department of Pathology, Metropolitan Hospital, Neon Faliron, 18547 Athens, Greece; 4 Department of Orthopaedics and Traumatology, “251” Hellenic Air Force General Hospital of Athens, 11525 Athens, Greece

**Keywords:** nivolumab, gastritis, melanoma, corticosteroids

## Abstract

Nivolumab, an anti-PD-1 check point inhibitor, is an immunotherapeutic agent, representing a major step in the treatment of melanoma. However, its use is associated with severe toxicities. Among them, gastrointestinal (GI) disorders from the lower GI tract have been widely reported. On the contrary, disorders from the upper GI tract are rare. Such a case of delayed nivolumab induced severe gastritis in a 53-year-old Caucasian female patient suffering metastatic melanoma is described. The patient’s symptoms from the upper GI tract began 4 months after nivolumab treatment initiation. The diagnosis was based on imaging, including PET/CT, endoscopical and pathological findings. The side effect was successfully treated with prolonged administration of proton pump inhibitors and corticosteroids. There are only a few cases of immune check point inhibitors (ICPis) induced upper GI tract disorders, while it seems that the symptoms from nivolumab induced upper GI tract damages appear later than those reported in the lower part. Nivolumab, among other side effects, may cause severe gastritis. Hence, this pathological entity should be included in the list of this drug’s side effects.

## 1. Introduction

Immunotherapy represents a major step in the management of melanoma, since it has improved dramatically responses to treatment and overall survival of patients suffering this malignancy. However, this treatment may be associated with severe, sometimes life threatening, toxicities [1,2].

Immune suppression caused by malignant cells and consequent evasion of healthy tissues are hallmarks of the cancer metastatic process. A series of co-inhibitory and co-stimulatory receptors, and their ligands, are involved, controlling these mechanisms such as the programmed cell death protein-1 (PD-1) and/or the programmed death-ligand-1 (PDL-1) axis, that stand out as therapeutic targets [3].

Nivolumab is a human IgG4 monoclonal antibody against PD-1, acting as an immune check point inhibitor (ICPi). ICPis block a signal, given by the tumor cells, that prevents activated T-cells from killing the malignant ones, thus allowing the immune system to attack the tumor. Nivolumab has been widely used successfully as monotherapy or in combination with other ICPis against several malignancies including melanoma [4].

The use of nivolumab, however, has been associated with a number of side effects, such as fever, immune related inflammation and dysfunction of several organs, namely pneumonitis, colitis, hepatitis, nephritis, hypo-and hyper-thyroidism, type I diabetes mellitus, iridocyclitis, neuropathies and skin toxicities. This drug’s gastrointestinal (GI) toxicities have been often observed and widely reported. However, gastritis has only recently been included in the reported GI toxicities of this agent [5,6]. Hence, there is a very limited number of nivolumab induced gastritis cases in the literature, despite the extensive use of this immunotherapeutic agent during the last decade, especially in the treatment of melanoma [2,5,6,7,8,9,10].

The case of an extremely rare nivolumab induced severe gastritis in a 53-year-old Caucasian woman with metastatic melanoma (MM) is presented. The role of PET CT in diagnosing this rare complication is also reported.

## 2. Case Presentation

A 53-year-old female patient suffering MM was hospitalized for progressive and gradually deteriorating loss of appetite, gastric discomfort and pain, nausea and vomiting during the two previous months, with consequent weight loss.

She had prior history of pharmaceutical iridocyclitis of the right eye and hypothyroidism, due to side effects of the BRAF and MECK inhibitors combination treatment she had received for her MM disease. At that point in time, she was on nivolumab monotherapy every two weeks, already for 6 months.

Her clinical examination, on admission, was unremarkable, except for mild sensitivity at the deep palpation of the gastric area. Her hematological and biochemical profiles were within normal limits.

**Figure 1:**
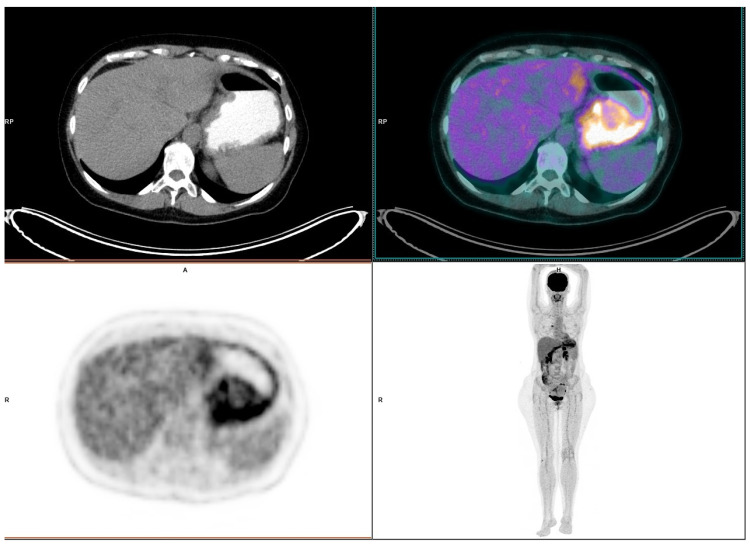
PET/CT, evaluating the metastatic melanoma status revealing intense metabolic uptake in the stomach.

A PET/CT, performed soon after her admission to the hospital, to evaluate the MM status and to restage her disease, showed intense metabolic uptake in the stomach area, indicating a malignant entity such as infiltration by MM, lymphoma or gastric carcinoma. Inflammation was also included in the differential diagnosis, taking into account the intense glucose metabolism of inflammatory cells [11] (Figure 1).

A consequent esophago-gastro-duodenoscopy (OGD) revealed severe erythematous gastritis, with frangibility, edema and mucous exudate, involving the antrum and the duodenal bulb with no ulcers seen macroscopically. Biopsies have been taken from the gastric mucosa.

**Figure 2:**
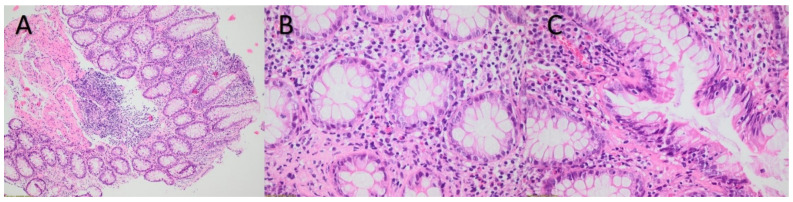
(**A**) Mucosa with normal architecture and mucin production showing moderate inflammatory infiltrate of the lamina propria. H-E stainx10. (**B**) Increased number of eosinophils in the lamina propria. H-E stainx40. (**C**) Extension of the eosinophils into the surface epithelium. H-E stainx40.

Histology of the biopsy specimens of the gastric and duodenal mucosa have shown moderate inflammatory infiltration of the lamina propria, increased number of eosinophils in the lamina propria and extension of the eosinophils into the surface epithelium, consistent with the diagnosis of severe gastritis (Figure 2).

She was started on proton pomp inhibitors (PPIs) and prednisolone at a dose of 0.5 mg/kg for 10 days with gradual improvement of her symptoms, starting six days after the initiation of this treatment.

She continued to receive PPIs and prednisolone in progressively decreasing doses for 3 months, with further, and finally complete, resolution of the symptoms from the upper GI tract.

A new gastroscopy performed three months later, while the nivolumab treatment had been discontinued, did not reveal signs of gastritis.

## 3. Discussion

A rare case of nivolumab-induced gastritis with its treatment and outcome is presented. The diagnosis has been based on endoscopical and pathological findings, while imaging (PET-CT), although is not included in the standard diagnostic process of gastritis, also indicated this rare complication in the present case.

Cumulative data from anti PDL-1/PD-1 treatments’ experience have shown that side effects from the GI tract occur often, reaching an incidence of 26% [1,2].

However, there are only a few case reports of ICPis induced upper GI tract disorders. In particular, there is a very limited number of nivolumab-associated upper GI tract toxicities reported in the English literature. Boike et al. reported esophagitis and gastritis after nivolumab treatment for malignant lymphoma, Kobayashi et al. hemorrhagic gastritis after nivolumab treatment for non-small cell lung cancer, Nguyin Tran et al. nivolumab-associated upper GI injury, Ojega Gomez et al. nivolumab induced neutrophilic gastritis, while Vindum et al. severe steroid refractory nivolumab induced gastritis, finally treated with infliximab [5,7,9,12,13].

The present patient’s gastroscopy revealed erythematous gastritis, severe edema and mucous exudate with no ulcers, while histology of the gastric mucosa has shown remarkable infiltration by eosinophiles. In particular the mucosa had normal architecture and mucin production. There was an inflammatory infiltration of the lamina propria with an increased number of eosinophiles, while eosinophiles were also extended into the surface of the epithelium. It is of note that other investigators have observed similar histological inflammatory findings induced by nivolumab and other ICPis with infiltration of the gastric mucosa by eosinophiles and also by neutrophils and/or by CD4, CD8 and CD20 lymphocytes [12,14].

PET/CT contributed, in this case, to the diagnosis of this extremely rare drug-related complication. In general, PET/CT has demonstrated to possess high diagnostic performance for the detection of soft-tissue, nodal and visceral metastases at initial staging or during follow-up, while this imaging examination represents a valuable tool for identification of tumor responses early in the course of several antineoplastic treatments. Furthermore, regarding immunotherapy, PET/CT has demonstrated the capability of assessing tumor response on a whole-body basis and detecting signs of immune activation as well as immune-related adverse effects [15,16]. In the reported case, the PET/CT, performed following the patient’s urgent admission to the hospital, revealed intense metabolic uptake in the stomach area, indicating a malignant entity such as infiltration by MM, lymphoma or gastric carcinoma, while inflammation was included in the differential diagnosis, however as a remote possibility. Further investigation with endoscopy and histological examination of specimens of the gastric mucosa established the diagnosis of severe gastritis.

It should be noted that even though PET/CT was used in the reported case, the diagnosis of gastritis is made through medical history and physical examination, while these findings are confirmed with esophago-gastro-duodenoscopy. The PET/CT was performed soon after the patient’s admission to the hospital in order to evaluate the MM status and to restage her disease.

ICPis associated lower GI tract toxicity, such as colitis, appearing six to nine weeks after initiation of treatment, has been widely observed and reported [1,2,6]. On the contrary, in the present case, symptoms from the upper GI tract occurred 6 months after the initiation of nivolumab treatment, a finding similar to those reported by Boike et al. and Kobayashi et al., describing upper GI disorders six and four months after the initiation of treatment, respectively [5,7]. The present case’s histological findings are particular, since mostly eosinophiles have infiltrated the mucosa and the surface of the epithelium, while in previous cases the infiltration was also characterized by eosinophiles, but mainly by neutrophiles and CD4, CD8 and CD20 lymphocytes [5,7,12,13,14].

This observation, along with the few previous reports, permits the assumption that symptoms from the upper GI tract nivolumab induced damages appear later than those reported of the lower part [1,2].

The mechanisms underlying these immune response adverse events remain unknown. It has been proposed that cell and tissue injury is due to self-reactive CD8 -positive T cells, and/or that the damage is probably caused by autoantibody production from CD4-positive T cells [14].

Steroids are the treatment of choice for the ICis induced GI toxicities, whereas the anti-tumor necrosis factors antibodies, infliximab and vedolizumab have been successfully used in severe and steroid-refractory gastritis and colitis cases [6,9,13].

Probably the incidence of autoimmune induced gastritis might be underestimated, since gastroscopy is not routinely performed in cases with upper GI symptoms such as nausea, vomiting, odynophagia, dysphagia and epigastric pain.

The present patient responded promptly and well six days after initiation of PPIs and steroid treatment, which was continued, in lower doses, for a prolonged time period.

## 4. Conclusions

In conclusion, nivolumab, among other side effects, may cause severe gastritis. Hence, gastritis should be included in the list of this drug’s side effects. Oncologists should have an increased index of suspicion for this complication, in patients treated with nivolumab. Even though PET/CT contributed to the diagnosis of the reported case, it should be noted that esophago-gastro-duodenoscopy represents the gold standard diagnostic approach for confirmation of this particular and severe side effect.

## Data Availability

Not applicable.

## References

[1] Gong J., Chehrazi-Raffle A., Reddi S., Salgia R. (2018). Development of PD-1 and PD-L1 inhibitors as a form of cancer immunotherapy: A comprehensive review of registration trials and future considerations. J. Immunother. Cancer.

[2] Darnell E.P., Mooradian M.J., Baruch E.N., Yilmaz M., Reynolds K.L. (2020). Immune-Related Adverse Events (irAEs): Diagnosis, Management, and Clinical Pearls. Curr. Oncol. Rep..

[3] Seidel J.A., Otsuka A., Kabashima K. (2018). Anti-PD-1 and Anti-CTLA-4 Therapies in Cancer. Mechanisms of Action, Efficacy, and Limitation. Front. Oncol..

[4] Rajan A., Kim C., Heery C.R., Guha U., Gulley J.L. (2016). Nivolumab, anti-programmed death-1 (PD-1) monoclonal antibody immunotherapy: Role in advanced cancers. Hum. Vaccin. Immunother..

[5] Boike J., Dejulio T. (2017). Severe Esophagitis and Gastritis from Nivolumab Therapy. ACG Case Rep. J..

[6] Puzanov I., Diab A., Abdallah K., Bingham C.O., Brogdon C., Dadu R., Hamad L., Kim S., Lacouture M., LeBoeuf N.R. (2017). Managing toxicities associated with immune checkpoint inhibitors: Consensus recommendations from the Society for Immunotherapy of Cancer (SITC) Toxicity. J. Immunother. Cancer.

[7] Kobayashi M., Yamaguchi O., Nagata K., Nonaka K., Ryozawa S. (2017). Acute hemorrhagic gastritis after nivolumab treatment. Gastrointest. Endosc..

[8] Ebisutani N., Tozawa K., Matsuda I., Nakamura K., Tamura A., Hara K., Kondo T., Terada T., Tomita T., Oshima T. (2021). A Case of Severe Acute Gastritis as an Immune-Related Adverse Event after Nivolumab Treatment: Endoscopic and Pathological Findings in Nivolumab-Related Gastritis. Dig Dis. Sci..

[9] Vindum H.H., Agnholt J.S., Nielsen A.W.M., Nielsen M.B., Schmidt H. (2020). Severe steroid refractory gastritis induced by Nivolumab: A case report. World J. Gastroenterol..

[10] Rovedatti L., Lenti M.V., Vanoli A., Feltri M., De Grazia F., Di Sabatino A. (2020). Nivolumab-associated active neutrophilic gastritis. J. Clin. Pathol..

[11] Wu C.X., Zhu Z.H. (2014). Diagnosis and evaluation of gastric cancer by positron emission tomography. World J. Gastroenterol..

[12] Ojeda Gómez A., Jiménez García N., Barragán Martínez J., García Soria A., Sáez Fuster J., Cabezas Macián M., García Sepulcre M.F. (2021). Acute neutrophilic gastritis induced by nivolumab used as treatment for non- metastatic malignant melanoma. Rev. Esp. Enferm. Dig..

[13] Nguyen Tran C., Abu-Sbeih H., Luo W., Lu Y., Wang Y. (2019). Vedolizumab Achieved Clinical and histologic remission in a patient with lung cancer who had a steroid--refractory upper gastrointestinal injury due to nivolumab treatment. J. Immunother. Precis. Onc..

[14] Onuki T., Morita E., Sakamoto N., Nagai Y., Sata M., Hagiwara K. (2018). Severe upper gastrointestinal disorders in pembrolizumab-treated non-small cell lung cancer patient. Respirol. Case Rep..

[15] Wong A.N.M., McArthur G.A., Hofman M.S., Hicks R.J. (2017). The advantages and challenges of using FDG PET/CT for response assessment in melanoma in the era of targeted agents and immunotherapy. Eur. J. Nucl. Med. Mol. Imaging.

[16] Aide N., Iravani A., Prigent K., Kottler D., Alipour R., Hicks R.J. (2022). PET/CT variants and pitfalls in malignant melanoma. Cancer Imaging.

